# Short-term Changes in Health-related Quality of Life of Patients Undergoing Radical Surgery for Upper Urinary Tract Urothelial Carcinoma: Results from a Prospective Phase 2 Clinical Trial

**DOI:** 10.1016/j.euros.2023.12.005

**Published:** 2024-01-05

**Authors:** Thomas van Doeveren, Sebastiaan Remmers, Vera Atema, Roderick C.N. van den Bergh, Egbert R. Boevé, Erik B. Cornel, Antoine G. van der Heijden, Kees Hendricksen, Evelyne C.C. Cauberg, Rens A.L. Jacobs, Bin K. Kroon, Annemarie M. Leliveld, Richard P. Meijer, Bob Merks, Jorg R. Oddens, Luc Roelofs, Diederik M. Somford, Peter de Vries, Bart Wijsman, Willemijn A.K.M. Windt, Peter J. Zwaan, Pim J. van Leeuwen, Joost L. Boormans, Katja K.H. Aben

**Affiliations:** a Department of Urology, Erasmus MC Cancer Institute, University Medical Centre Rotterdam, Rotterdam, The Netherlands; b Department of Research and Development, Netherlands Comprehensive Cancer Organisation, Utrecht, The Netherlands; c Department of Urology, St. Antonius Ziekenhuis, Nieuwegein, The Netherlands; d Department of Urology, Franciscus Gasthuis en Vlietland, Rotterdam, The Netherlands; e Department of Urology, Ziekenhuis Groep Twente, Hengelo, The Netherlands; f Department of Urology, Radboud University Medical Center, Nijmegen, The Netherlands; g Department of Urology, Netherlands Cancer Institute, Amsterdam, The Netherlands; h Department of Urology, Isala Medical Center, Zwolle, The Netherlands; i Department of Urology, Zuyderland Medical Center, Heerlen and Sittard, The Netherlands; j Department of Urology, Rijnstate Medical Center, Arnhem, The Netherlands; k Department of Urology, University Medical Center Groningen, Groningen, The Netherlands; l Department of Urology, University Medical Center Utrecht, Utrecht, The Netherlands; m Department of Urology, Haaglanden Medical Center, Leidschendam, The Netherlands; n Department of Urology, AmsterdamUMC, University of Amsterdam, Amsterdam, The Netherlands; o Department of Urology, Treant Zorggroep, Emmen, The Netherlands; p Department of Urology, Canisius Wilhelmina Ziekenhuis, Nijmegen, The Netherlands; q Department of Urology, Elisabeth-Tweesteden Medical Center, Tilburg, The Netherlands; r Department of Urology, Martini Ziekenhuis, Groningen, The Netherlands; s Department of Urology, Gelre Ziekenhuis, Apeldoorn, The Netherlands; t Department for Health Evidence, Radboud University Medical Center, Nijmegen, The Netherlands

**Keywords:** European Organisation for Research and Treatment of Cancer Quality of Life Questionnaire C30, Health-related quality of life, Radical surgery, Short term, Upper urinary tract, Urothelial carcinoma

## Abstract

**Background and objective:**

The possible negative impact of radical surgery on patients’ health-related quality of life (HRQoL) plays an important role in preoperative counseling. Here, we analyzed the HRQoL of patients treated for upper urinary tract urothelial carcinoma (UTUC) in the context of a single-arm phase 2 multicenter study, in which the safety and efficacy of a single preoperative intravesical instillation with mitomycin C were investigated. Our objective was to investigate early changes in HRQoL in patients undergoing radical surgery for UTUC and identify factors associated with these outcomes.

**Methods:**

Patients with pTanyN0-1M0 UTUC were prospectively included. HRQoL was assessed using the European Organisation for Research and Treatment of Cancer Quality of Life Questionnaire C30 (EORTC QLQ-C30) questionnaire at baseline, and at 1 and 3 mo after surgery. A linear mixed model was used to evaluate the changes in HRQoL over time and identify the variables associated with these outcomes. The clinical effect size was used to assess the clinical impact and level of perceptibility of HRQoL changes for clinicians and/or patients based on given thresholds.

**Key findings and limitations:**

Between 2017 and 2020, 186 patients were included. At baseline, 1 mo after surgery, and 3 mo after surgery, response rates were 91%, 84%, and 78%, respectively. One month after surgery, a statistically significant and clinically relevant deterioration was observed in physical, role, and social functioning, and for the included symptom scales: constipation, fatigue, and pain. An improvement in emotional functioning was observed. At 3 mo, HRQoL returned to baseline levels, except emotional functioning, which improved at 1 mo and persisted to be better than that before surgery. Age >70 yr was associated with worse physical functioning, but better social and emotional functioning. Male patients reported better emotional functioning than females. Postoperative complications were negatively associated with social functioning.

**Conclusions and clinical implications:**

UTUC patients treated with radical surgery experienced a significant, albeit temporary, decline in HRQoL. Three months following surgery, HRQoL outcomes returned to baseline levels. This information can be used to counsel UTUC patients before undergoing radical surgery and contextualize recovery after surgery.

**Patient summary:**

We investigated the changes in quality of life as reported by patients who underwent surgery for upper tract urothelial carcinoma (UTUC). We found that patients experienced a decline in quality of life 1 mo after surgery, but this was temporary, with full recovery of quality of life 3 mo after surgery. These findings can help doctors and other medical staff in counseling UTUC patients before undergoing radical surgery.

## Take Home Message

A temporary decline in patient-reported health-related quality of life can be expected following radical surgery for upper tract urothelial carcinoma, with full recovery to presurgery levels 3 mo after surgery.

## 1 Introduction

Urothelial carcinoma predominantly originates in the urinary bladder, but in 5–10% of patients, the upper urinary tract, that is, the ureter or renal pelvis, is the primary site of origin [1]. The incidence of upper tract urothelial carcinoma (UTUC) is on the rise in multiple countries [2], [3], [4]. In The Netherlands, the age-standardized incidence rate increased from two cases per 100 000 persons per year in 1993 to over three cases per 100 000 persons per year in 2017 [4]. Although kidney-sparing surgery is a treatment option in selected low-risk UTUC patients, the European Association of Urology (EAU) UTUC guideline recommends radical nephroureterectomy (RNU) with ipsilateral bladder-cuff excision for localized UTUC [1]. Following RNU, patients undergo close surveillance, although adjuvant chemotherapy can be considered for locally advanced UTUC (pathological stage T3/T4) [5], [6]. Therefore, surgery and subsequent treatment trajectory can significantly impact patients’ quality of life (QoL).

The Peri-Operative chemotherapy versus sUrveillance in upper Tract urothelial cancer (POUT) trial is the only study to have reported on the health-related quality of life (HRQoL) for UTUC patients treated with RNU. In this trial, UTUC patients received RNU with or without adjuvant chemotherapy [5]. The mean global health status scores as measured by the European Organisation for Research and Treatment of Cancer (EORTC) Quality of Life Questionnaire (QLQ)-C30 were reported at baseline (ie, shortly after the surgery) and after 3, 6, 12, and 24 mo. In UTUC patients treated by RNU, but without adjuvant chemotherapy, no clear changes were observed in the time period between RNU and 3 mo later. In UTUC patients receiving adjuvant chemotherapy, the mean global health score deteriorated significantly during and after chemotherapy up to 6 mo after baseline. No results were reported on the impact of surgery on other scales of the EORTC QLQ-C30 (social, cognitive, physical, role, and emotional functioning), nor were factors evaluated that may affect global health status.

Given the evident lack of literature considering the impact of radical surgery on HRQoL outcomes in UTUC patients undergoing RNU, we aimed to assess the impact of surgery on HRQoL and identify factors associated with changes in HRQoL outcomes in UTUC patients.

## 2 Patients and methods

### 2.1 Study design

Patients diagnosed with UTUC between 2017 and 2020 and treated with radical surgery were included in the REBACARE trial, a single-arm multicenter study (EU Clinical Trials Register; EudraCT number 2017-000949-53). Study details have been published previously [7]. Adults (age ≥18 yr) diagnosed with primary cTanyN0-1M0 UTUC without receiving neoadjuvant chemotherapy and without (a history of) bladder cancer were enrolled between November 2017 and July 2020 in 18 hospitals in The Netherlands. The majority of patients received a single preoperative intravesical instillation with mitomycin C (MMC; intention-to-treat protocol) within 3 h before RNU or partial ureterectomy with bladder cuff excision instead of a postoperative intravesical instillation, which is the standard of care [8]. Patient, tumor, and treatment characteristics were collected prospectively. The primary endpoint was the proportion of histologically proven intravesical recurrences 2 yr after surgery. The secondary endpoint was the assessment of HRQoL by the EORTC QLQ-C30 at three points in time: at the time of inclusion (baseline; following the diagnosis of UTUC but prior to surgery), and 1 and 3 mo after surgery. Hard-copy questionnaires were used at baseline. Online questionnaires were used at 1 and 3 mo after surgery for which patients were invited by an e-mail. A varying time window of 2 wk was allowed for each measurement. Completed questionnaires (hard copy and online questionnaires) were processed and linked to the corresponding patient by data managers of The Netherlands Comprehensive Cancer Organisation (IKNL). The study was approved by the institutional review board of the Erasmus Medical Center and received enforceability permission for all participating sites (METC 2017-227 NL60919.078.17). The REBACARE trial was undertaken according to the principles of Good Clinical Practice and sponsored by the Dutch Cancer Society (KWF; project number 10319).

### 2.2 EORTC QLQ-C30

The validated EORTC QLQ-C30 version 3.0 was used to assess HRQoL [9], [10]. EORTC QLQ-C30 is a tool used widely to assess HRQoL in cancer patients, with 30 items covering different QoL scales; one scale assesses the global health status and five functional scales measure physical, role, emotional, cognitive, and social functioning. Three symptom scales measure the burden of fatigue, pain, and nausea/vomiting. In addition, six single items assess cancer-related symptoms, such as dyspnea, sleeping problems, appetite loss, constipation, diarrhea, and financial difficulties. All items are scored on a 4-point scale, ranging from “not at all” to “very much,” except for the global health score, which has a 7-point scale, ranging from “very poor” to “excellent”. All scores are transformed linearly to a 0–100 scale. For the global health score and functional scales, a higher score indicates better functioning, whereas for the symptom scales, a higher score indicates a higher symptom burden. Missing data were imputed according to the EORTC guidelines, provided that at least half of the items in that specific scale were completed [11].

Based on expert opinion and the expected minimal effect of the surgical intervention on dyspnea, sleep problems, appetite loss, nausea/vomiting, diarrhea, and financial difficulties, these symptom scales were not evaluated in the current study.

### 2.3 Statistical analyses

Descriptive analyses provide insight into the patient, tumor, and treatment characteristics. Data are presented separately for all included patients of the REBACARE trial, full responders, and responders who completed two of the three questionnaires. Categorical characteristics were described using frequencies (*n*) and percentages (%), and continuous variables were described using means and standard deviations or medians and interquartile ranges. All statistical analyses were performed using SPSS version 28.0.1.0 (SPSS Inc., Chicago, IL, USA) and R version 4.2.1 (R Foundation for Statistical Analysis, Vienna, Austria).

Longitudinal linear mixed model analyses assessed HRQoL changes over time for all five functional scales, the global health scale, and the three included symptom scales. All analyses were adjusted for predefined confounders, including sex (male vs female), age at diagnosis (reference 70 yr), age-adjusted Charlson Comorbidity Index, lymph node dissection (yes/no), pathological tumor stage (pT stage <pT2 vs ≥pT2), type of surgery (open vs laparoscopic/robot assisted), and surgical complications (yes [any degree of the Clavien-Dindo classification]/no) [12]. Since all questionnaires were collected at the same follow-up scheme, time was used as a categorical variable [13]. The model included baseline scores, as well as the scores at 1 and 3 mo. To adjust for clustering within patients, each individual patient was included as a random intercept. Longitudinal linear mixed model analyses correct for missing responses at random [13]. The effect size on HRQoL for all predefined confounders is presented separately, including the beta-coefficient, the 95% confidence intervals (CIs), and *p* values. Additionally, the interaction between “time (as a categorical variable) × sex” and “time × age” was included in the model as interaction terms to assess whether sex and/or age had different effects on HRQoL at different points in time (effect modification) [13].

To assess clinical relevance, clinical effect size (CES) was used to evaluate the impact of (statistically significant) differences. CES is calculated by the change in the mean score for the functional, global health, and symptom scales between baseline and 1 and 3 mo, and is categorized as trivial, small, medium, and large improvement/deterioration. The outcomes of CES are based on the thresholds suggested by the “Guidelines of interpretation of longitudinal QoL differences” by Cocks et al. [14]. This approach considers whether the impact on each HRQoL scale is perceptible for patients and/or clinicians apart from solely statistical significance (Supplementary Table 1) [14], [15].

## 3 Results

**Fig. 1:**
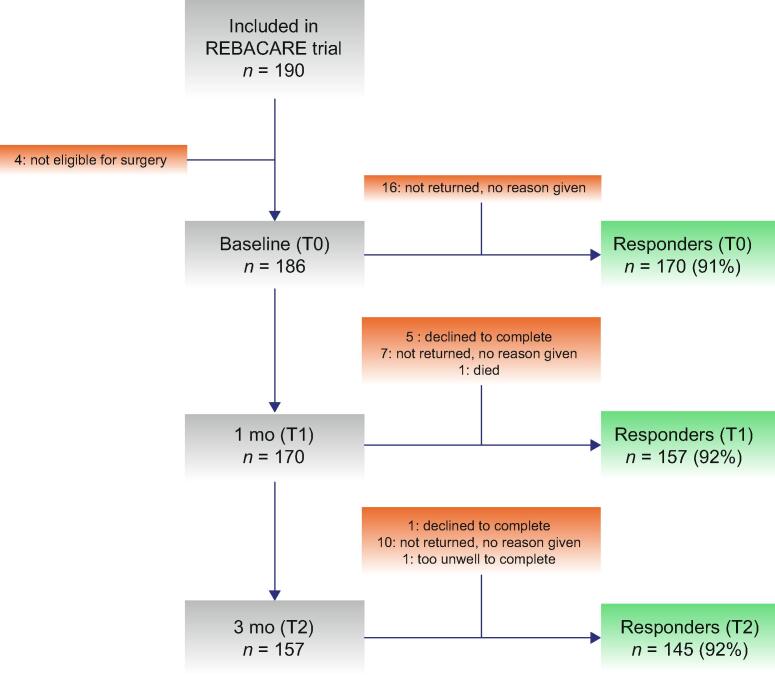
Flow diagram of the REBACARE HRQoL study. Responders answered all the questions. HRQoL = health-related quality of life.

In total, 190 patients diagnosed with primary nonmetastatic UTUC were enrolled in the REBACARE trial. Of these patients, 186 underwent radical surgery and 171 (92%) eventually received a preoperative intravesical instillation with MMC as part of the trial. The baseline questionnaire was completed by 170 (91%) patients. See Figure 1 for the flow chart of the REBACARE trial, including response rates. Table 1 presents the baseline characteristics of all surgically treated patients in the REBACARE trial, responders who completed all questionnaires (*N* = 133), and responders who completed at least two questionnaires (*N* = 157). No significant differences were observed in patient, tumor, and treatment characteristics between the three different groups, except for one: full responders were more likely to be diagnosed with a pT3 tumor (33% vs 28–29%) and less likely with pT1 tumors (14% vs 17–18%).

**Table 1:** Baseline characteristics of all surgically treated patients included in the REBACARE trial, the full responders, and the responders who filled in at least two of the three European Organisation for Research and Treatment of Cancer Quality of Life Questionnaire C30

Characteristic	REBACARE-trial^a^	Responders completed all three questionnaires	Responders with at least two questionnaires
	*N* = 186	*N* = 133	*N* = 157
Sex, *n* (%)			
Female	60 (32)	41 (31)	48 (31)
Age (yr)			
Mean (SD)	68.3 (9.1)	68.9 (8.6)	68.4 (9.0)
WHO performance status, *n* (%)			
0	145 (78)	108 (81)	127 (81)
1	34 (18)	20 (15)	24 (15)
2	3 (1.6)	3 (2.3)	3 (1.9)
Unknown	4 (2.2)	2 (1.5)	3 (1.9)
Charlson Comorbidity Index, *n* (%)			
Median (IQR)	6.0 (4–7)	6.0 (4–7)	6.0 (4–7)
≤4	57 (31)	41 (31)	50 (32)
>4	129 (69)	92 (69)	107 (68)
Type of surgery, *n* (%)			
RNU, open	23 (13)	17 (13)	19 (12)
RNU, laparoscopic/robot	153 (82)	108 (81)	130 (83)
Distal ureterectomy, open	6 (3.2)	4 (3.0)	4 (2.5)
Distal ureterectomy, laparoscopic/robot	4 (2.2)	4 (3.0)	4 (2.5)
Preoperative intravesical instillation with MMC, *n* (%)	171 (92)	124 (93)	144 (92)
Days of hospitalization, median (min–max)	9 (1–17)	8 (1–16)	9 (1–17)
Lymph node dissection, *n* (%)			
Yes	35 (19)	27 (20)	29 (19)
No	146 (78)	105 (79)	125 (80)
Unknown	5 (2.7)	1 (0.8)	3 (1.9)
Pathological tumor stage, *n* (%)			
Tis	4 (2.2)	3 (2.3)	4 (2.5)
Ta	63 (34)	43 (32)	52 (33)
T1	34 (18)	19 (14)	26 (17)
T2	25 (13)	20 (15)	24 (15)
T3	52 (28)	44 (33)	46 (29)
T4	5 (2.7)	3 (2.3)	4 (2.5)
pTx	3 (1.6)	1 (0.8)	1 (0.6)
Tumor grade (WHO 1973), *n* (%)			
Grade 1	19 (10)	13 (10)	16 (10)
Grade 2	72 (39)	51 (38)	60 (38)
Grade 3	84 (45)	61 (46)	72 (46)
Unknown	11 (5.9)	8 (6.0)	9 (5.7)
Lymph node involvement, *n* (%)			
No	27 (15)	21 (16)	22 (14)
Yes	8 (4.3)	6 (4.6)	7 (4.7)
pNx	151 (82)	106 (80)	128 (82)
Patients with a surgical complication (<30 d),^b^*n* (%)	59 (32)	42 (31)	52 (33)
Grade I	54 (29)	44 (33)	52 (33)
Grade II	12 (6.5)	5 (3.8)	9 (5.7)
Grade III	14 (7.5)	10 (7.5)	11 (7.0)
Grade IV	1 (0.5)	1 (0.8)	1 (0.6)
Grade V	1 (0.5)	–	–
Readmission rate after surgery (<30 d), *n* (%)	18 (10)	12 (9)	16 (10)

IQR = inter quartile range; MMC = mitomycin C; RNU = radical nephroureterectomy; SD = standard deviation; WHO = World Health Organization.

a Surgically treated.

b Some patients had multiple surgical complications.

### 3.1 Changes over time in HRQoL

At 1 mo following surgery, the global health status and cognitive functioning did not differ statistically from the average baseline score: –4.0 points (95% CI –9.3 to 1.3; CES small) and –3.8 points (95% CI –9.1 to 1.5; CES small). However, physical (–16.5 points, 95% CI –21.4 to –11.7, *p* < 0.001), role (–28.8 points, 95% CI –37.7 to –20.0, *p* < 0.001), and social functioning (–12.5 points, 95% CI –18.8 to –6.2, *p* < 0.001) deteriorated significantly compared with baseline (Table 2), and for these scales, medium to large CES was noted. Additionally, patients reported higher emotional functioning scores at 1 mo than at baseline, an improvement of 6.8 points (95% CI 1.2–12.4, *p* = 0.017) that was considered of small clinical relevance. Symptom scales showed that fatigue, pain, and constipation scores were higher at 1 mo than at baseline (*p* > 0.001, medium to large CES). At 3 mo after surgery, all functioning and symptom scales had returned to baseline levels except for the improvement in emotional functioning, which persisted (9.5 points, 95% CI 3.9–15.2, *p* = 0.001) and was of medium clinical relevance.

**Table 2:** Changes in the functioning scales of the European Organisation for Research and Treatment of Cancer Quality of Life Questionnaire C30 from baseline to 1 and 3 mo after radical surgery for UTUC

EORTC QLQ-C30	Mean	Change a	95% CI	*p* value	CES
Global health status					
Baseline	73.8				
After 1 mo	69.8	–4.0	(–9.3, 1.3)	0.14	Small
After 3 mo	77.4	3.6	(–1.8, 9.0)	0.19	Trivial
Physical functioning					
Baseline	81.8				
After 1 mo	65.3	–16.5	(–21.4, –11.7)	**<0.001**	Medium
After 3 mo	79.2	–2.8	(–7.5, 2.3)	0.30	Trivial
Role functioning					
Baseline	78.5				
After 1 mo	49.7	–28.8	(–37.7, –20.0)	**<0.001**	Large
After 3 mo	75.8	–2.8	(–11.8, 6.2)	0.5	Trivial
Social functioning					
Baseline	86.7				
After 1 mo	74.2	–12.5	(–18.8, –6.2)	**<0.001**	Medium
After 3 mo	87.0	0.3	(–6.1, 6.7)	>0.9	Trivial
Emotional functioning					
Baseline	70.9				
After 1 mo	77.7	6.8	(1.2, 12.4)	**0.017**	Small
After 3 mo	80.4	9.5	(3.9, 15.2)	**0.001**	Medium
Cognitive functioning					
Baseline	85.2				
After 1 mo	81.4	–3.8	(–9.1, 1.5)	0.16	Small
After 3 mo	88.5	3.3	(–2.1, 8.7)	0.22	Small
Fatigue					
Baseline	27.1				
After 1 mo	44.5	17.4	(10.9, 24.0)	**<0.001**	Medium
After 3 mo	25.3	–1.8	(–8.5, 4.9)	0.6	Trivial
Pain					
Baseline	19.5				
After 1 mo	38.3	18.9	(11.4, 26.4)	**<0.001**	Large
After 3 mo	19.9	0.5	(–7.2, 8.1)	0.9	Trivial
Constipation					
Baseline	6.4				
After 1 mo	22.5	16.1	(7.7, 24.6)	**<0.001**	Medium
After 3 mo	8.3	1.9	(–6.7, 10.5)	0.7	Trivial

CCI = Charlson Comorbidity Index; CES = clinical effect size; CI = confidence interval; EORTC QLQ-30 = European Organisation for Research and Treatment of Cancer Quality of Life Questionnaire C30; HRQoL = health-related quality of life; UTUC = upper tract urothelial carcinoma.

The *p* values of <0.05 are considered significant; HRQoL subscales range from 0 to 100.

The clinical effect size is shown as trivial, small, medium, or large, based on the thresholds as indicated by Cocks et al. [14]. Scores were adjusted for age, pT stage, age-adjusted CCI, surgical complication, type of surgery, and lymph node dissection using a linear mixed model analysis.

a Difference between mean baseline score and mean defined time point.

### 3.2 Patient, tumor, and treatment-related factors and HRQoL

The results of the longitudinal linear mixed model analyses, excluding the time variable (interaction terms), are presented in Table 3. Age was found to be associated with better social and emotional functioning, but worse physical functioning. Men reported better emotional functioning, while a surgical complication (any degree vs no surgical complication) had a negative impact on social functioning. No significant associations were observed for pathological T stage (<pT2 vs ≥pT2), Charlson Comorbidity Index (≤4 vs >4), lymph node dissection (yes vs no), and type of surgery (open vs laparoscopic). Furthermore, no significant associations were found for patient-, tumor-, and treatment-related factors with the different symptom scales.

**Table 3:** Associations between patient, treatment, and tumor characteristics and the functional scales of the European Organisation for Research and Treatment of Cancer Quality of Life Questionnaire C30 during 3 mo following radical surgery for UTUC using a linear mixed model analysis

EORTC QLQ-C30 Functional scales	Global health status			Physical functioning			Role functioning			Social functioning			Emotional functioning			Cognitive functioning		
	B	95% CI	*p* value	B	95% CI	*p* value	B	95% CI	*p* value	B	95% CI	*p* value	B	95% CI	*p* value	B	95% CI	*p* value
Age (reference 70 yr)	0.1	(–0.2, 0.5)	0.4	–0.3	(–0.6, –0.02)	**0.035**	0.1	(–0.3, 0.6)	0.6	0.5	(0.1, 0.9)	**0.017**	0.5	(0.1, 0.9)	**0.012**	0.2	(–0.1, 0.5)	0.22
Sex																		
Female		Reference			Reference			Reference			Reference			Reference			Reference	
Male	1.5	(–4.6, 7.5)	0.6	3.9	(–1.6, 9.4)	0.17	2.3	(–6.4, 10.9)	0.6	2.2	(–4.9, 9.2)	0.6	9.3	(2.6, 16.0)	**0.007**	5.7	(–0.02, 11.5)	0.052
pT stage																		
<pT2		Reference			Reference			Reference			Reference			Reference			Reference	
≥pT2	–2.3	(–7.0, 2.3)	0.3	–1.2	(–5.4, 3.0)	0.6	–1.3	(–7.4, 4.8)	0.6	–2.5	(–7.9, 2.9)	0.4	–2.5	(–7.7, 2.8)	0.4	1.6	(–2.7, 5.9)	0.5
CCI																		
≤4		Reference			Reference			Reference			Reference			Reference			Reference	
>4	–0.03	(–5.4, 5.4)	>0.9	–0.6	(–5.5, 4.2)	0.8	0.2	(–6.8, 7.2)	>0.9	0.4	(–5.8, 6.7)	>0.9	0.5	(–5.6, 6.6)	0.9	–0.5	(–5.5, 4.5)	0.9
Type of surgery																		
Open		Reference			Reference			Reference			Reference			Reference			Reference	
Laparoscopic/robot	2.0	(–4.5, 8.5)	0.6	1.7	(–4.2, 7.6)	0.6	3.7	(–4.8, 12.1)	0.4	3.4	(–4.1, 10.9)	0.4	2.7	(–4.7, 10.0)	0.5	–1.0	(–7.1, 5.0)	0.7
Lymph node dissection																		
No		Reference			Reference			Reference			Reference			Reference			Reference	
Yes	–2.0	(–7.3, 3.3)	0.5	0.1	(–4.7, 4.9)	>0.9	0.9	(–7.1, 8.9)	0.8	1.1	(–5.1, 7.3)	0.7	–2.1	(–7.7, 3.5)	0.5	–3.2	(–8.4, 1.9)	0.22
Surgical complication																		
No		Reference			Reference			Reference			Reference			Reference			Reference	
Yes	–2.7	(–7.2, 1.9)	0.25	–2.5	(–6.6, 1.6)	0.24	–3.2	(–10.1, 3.7)	0.4	–6.3	(–11.6, –0.9)	**0.022**	0.7	(–4.2, 5.5)	0.8	–1.0	(–5.4, 3.4)	0.7

CCI = Charlson Comorbidity Index; CI = Confidence Interval; UTUC = upper tract urothelial carcinoma.

The *p* values of <0.05 were considered statistically significant.

**Fig. 2:**
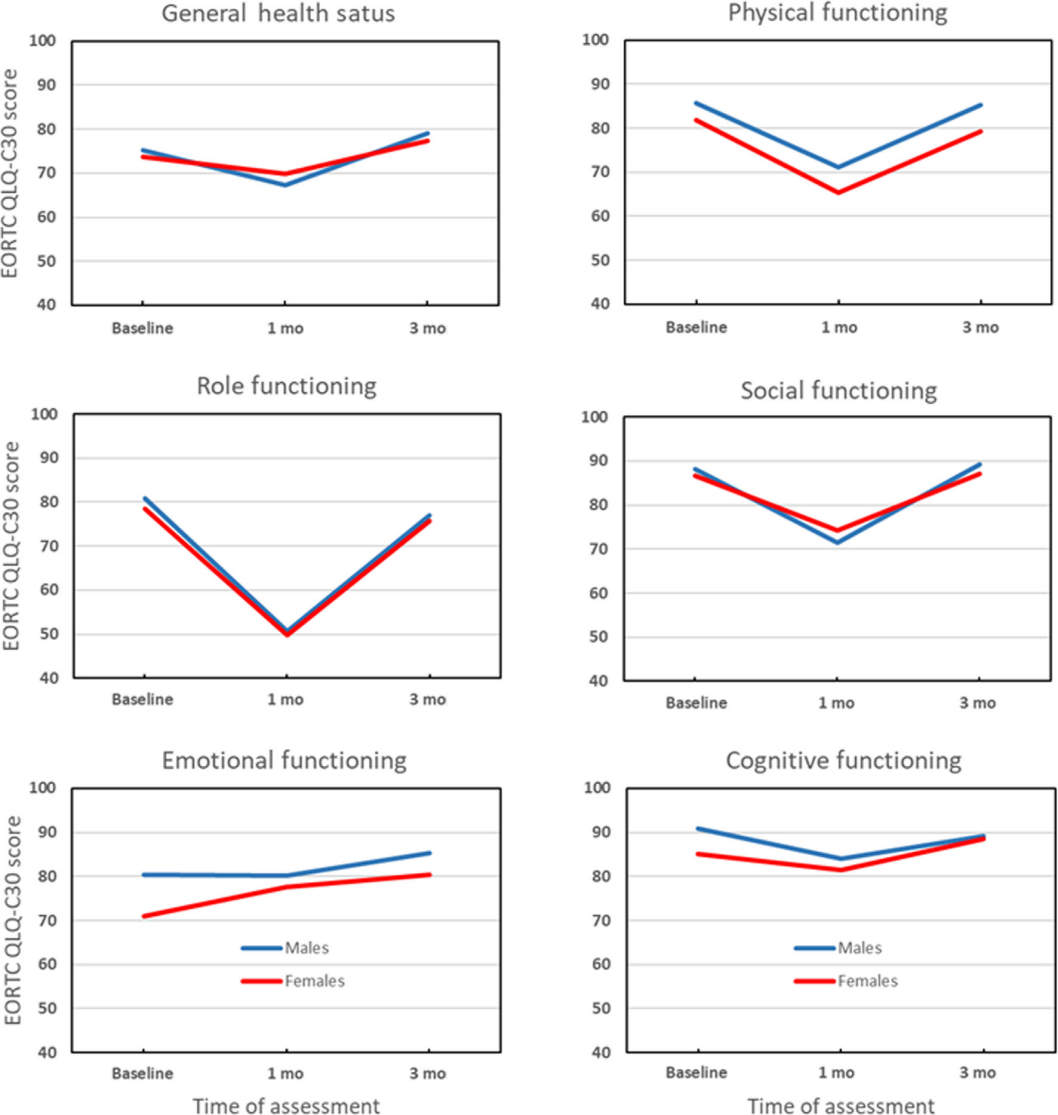
Mean scores at baseline, and at 1 and 3 mo after radical surgery for all functional scales of the European Organisation for Research and Treatment of Cancer Quality of Life Questionnaire C30 for male versus female UTUC patients. Scores were adjusted for age, pT stage, age-adjusted CCI, surgical complication, type of surgery, and lymph node dissection using a linear mixed model analysis. CCI = Charlson Comorbidity Index; EORTC QLQ-C30 = European Organisation for Research and Treatment of Cancer Quality of Life Questionnaire C30; UTUC = upper tract urothelial carcinoma.

Sex was the only observed effect modifier, as females experienced a significantly greater improvement in emotional functioning from baseline to 1 mo following surgery than men (Fig. 2). No effect modification for sex was noted in other HRQoL scales.

## 4 Discussion

This study showed that patients with UTUC who underwent radical surgery, preceded by an intravesical instillation with MMC for most patients, experienced a temporary decline in physical, role, and social functioning, but all scores returned to the pretreatment levels at 3 mo after surgery. Similar results were found for fatigue, pain, and constipation. However, for emotional functioning, an improvement was observed at 1 mo, which persisted at 3 mo after surgery. Older patients experienced better social and emotional functioning but worse physical functioning. Male sex was associated with greater emotional well-being, and surgical complications compromised social functioning.

A comparison between the current REBACARE trial and the POUT trial is challenging due to the difference in the timing of the baseline assessment of the QLQ-C30. In the POUT trial, the assessment was carried out shortly after surgery, while in the REBACARE trial, it was conducted prior to surgery. Nevertheless, for the group of patients who received RNU only, no clear changes during the period shortly after and up to 3 mo following RNU were noted for the global health status, which is consistent with the trend found in our study [5]. In the POUT trial, however, the global health status deteriorated again after 3 mo in the POUT trial. An explanation for this observation could be that only patients with advanced UTUC who had a high risk of disease progression were included in this trial. Previous studies have shown that an advanced disease stage has a negative impact on HRQoL [7], [16], [17]. High dropout rates over time and disease progression may also have contributed to the observed deterioration in global health status. Based on these findings, it is important to be aware of a potential decline in HRQoL of UTUC patients >3 mo after surgery. However, the observed rapid recovery in patient-reported QoL in both the POUT and the REBACARE trial demonstrate that the administration of adjuvant chemotherapy, which is recommended by the EAU guideline for a subgroup of UTUC patients, seems feasible [1].

Patients included in our study showed a statistically significant and clinically relevant improvement in emotional functioning over time. This contrasts with the results concerning the other functional scales for which only temporary effects within the study period were observed. The improvement in emotional functioning might reflect reduced anxiety due to surgical eradication of the tumor, as described in previous studies evaluating oncological surgery [11], [18]. The timing of the first assessment, conducted shortly after diagnosis, may have amplified this effect, as the initial diagnosis of UTUC could have caused an immediate deterioration in emotional functioning.

We found that females scored lower on emotional functioning at baseline and at 1 and 3 mo after surgery than men. This difference is consistent with other cancer populations [19], [20], [21]. Varying results have been reported on gender disparities with regard to coping and anxiety or depression after surgery for multiple malignancies [22], [23]. For most cancers, female patients tend to experience more anxiety or depression following diagnosis and treatment [24]. Notably, in our study, females tend to experience a greater improvement in emotional functioning during the period from before surgery to 1 mo after surgery than men. Although the exact reason for this observation remains unknown, it is important to further investigate this finding. It may have implications for counseling female UTUC patients on the possibility of significant emotional recovery following surgery.

Patients who experienced a surgical complication within the 1st month following radical surgery for UTUC showed a decline in their social functioning. This is consistent with a study by Brown et al. [25] on patients with colorectal cancer who underwent surgery, which reported a negative impact of surgical complications on social functioning. In this study, patients with complications had significantly lower social functioning scores at 3 mo after surgery, which persisted up to 36 mo. The reasons for this effect may include longer hospital stay, additional interventions or medication, slower recovery, and psychological or physical consequences. Clinicians should take note of these potential long-term effects of surgical complications on the HRQoL of surgically treated patients.

This study is, to our knowledge, the first to report on the impact of radical surgery for UTUC on multiple scales of HRQoL and potential confounders associated with these outcomes. As the incidence of UTUC and consequently the number of radical surgeries increases, understanding the patient-reported QoL after surgery becomes essential to enhance shared decision-making and monitor UTUC patients in daily clinical practice [26], [27]. This understanding can be used to inform patients before undergoing surgery and to contextualize their recovery after surgery. Moreover, it may help align the expectations of patients and surgeons as they often have differing assumptions regarding the impact of surgery on HRQoL [28], [29].

The present study has several limitations that should be acknowledged. First, due to the design of the REBACARE trial, patients with node-positive (>pN1) or distant metastatic UTUC were excluded. Therefore, the outcomes of our study cannot be generalized to UTUC patients with metastatic disease. Second, although the compliance rate for completed questionnaires at baseline was high (91%), only 72% of the patients completed the questionnaires at all three assessment points. As the reasons for nonresponse are largely unknown, it is possible that patients selectively dropped out, which could introduce a bias in our results. Finally, we did not differentiate the degree of surgical complications within the linear mixed model analysis, making it unclear how much the effect on HRQoL is attributable to patients with more severe (higher Clavien-Dindo grade) surgical complications.

## 5 Conclusions

Patients undergoing radical surgery for UTUC experience a temporary deterioration in most HRQoL scales shortly after surgery, with full recovery observed at 3 mo after surgery. An improvement was observed in emotional well-being. These findings can help clinicians counsel patients about the expected impact of radical surgery for UTUC on HRQoL and identify patients at a risk of impaired recovery of their QoL. Considering the EAU’s recommendation for adjuvant treatment following surgery, this study suggests that, for the majority of eligible patients, HRQoL will be satisfactory with this treatment approach.

  ***Author contributions:*** Thomas van Doeveren had full access to all the data in the study and takes responsibility for the integrity of the data and the accuracy of the data analysis.

  *Study concept and design*: van Doeveren, Boormans, van Leeuwen, Aben, Remmers, Atema.

*Acquisition of data*: van Doeveren, van den Bergh, Boevé, Cornel, van der Heijden, Hendricksen, Cauberg, Jacobs, Kroon, Leliveld, Meijer, Merks, Oddens, Roelofs, Somford, de Vries, Wijsman, Windt, Zwaan, Boormans.

*Analysis and interpretation of data*: van Doeveren, Boormans, van Leeuwen, Aben, Remmers, Atema.

*Drafting of the manuscript*: van Doeveren, Boormans, van Leeuwen, Aben, Remmers, Atema.

*Critical revision of the manuscript for important intellectual content*: All authors.

*Statistical analysis*: van Doeveren, Remmers, Atema.

*Obtaining funding*: Boormans, van Leeuwen, Aben.

*Administrative, technical, or material support*: van Doeveren.

*Supervision*: Boormans, van Leeuwen, Aben.

*Other*: None.

  ***Financial disclosures:*** Thomas van Doeveren certifies that all conflicts of interest, including specific financial interests and relationships and affiliations relevant to the subject matter or materials discussed in the manuscript (eg, employment/affiliation, grants or funding, consultancies, honoraria, stock ownership or options, expert testimony, royalties, or patents filed, received, or pending), are the following: None.

  ***Funding/Support and role of the sponsor:*** This work was supported by the Dutch Cancer Society (KWF).

  ***Acknowledgments:*** We thank the patients who participated in this trial and staff at the participating centers and at the IKNL, especially Jessica van Raaij, Margriet van Hövell, and Joline Claassen; we also thank Marjan de Jong and Rogier Pullens for monitoring the REBACARE trial.
