# Supplementary material for: Short-term Changes in Health-related Quality of Life of Patients Undergoing Radical Surgery for Upper Urinary Tract Urothelial Carcinoma: Results from a Prospective Phase 2 Clinical Trial
Source: Eur Urol Open Sci. 2024 Jan 5;60:15–23. doi: 10.1016/j.euros.2023.12.005 (PMC10874848; doi:10.1016/j.euros.2023.12.005)
Supplement: Supplementary Table 1 [file mmc1.docx]

**Supplementary Table 1:** Scores at baseline, one, and three months after radical surgery for UTUC and the associations between patient, tumor, and treatment characteristics of the symptom scales constipation, fatigue, and pain of the *European Organisation for Research and Treatment of Cancer Quality of Life Questionnaire*-C30. The clinical effect size, shown as trivial, small, medium, or large based on the thresholds as indicated by *Cocks et al.* [1]. Scores were adjusted for age, pT-stage, age-adjusted CCI, surgical complication, type of surgery, and lymph node dissection using a linear mixed model analysis.

| **Supplementary Table 1.** | | | | | | | | | | | | |
| --- | --- | --- | --- | --- | --- | --- | --- | --- | --- | --- | --- | --- |
| EORTC QLQ-C30  *Symptom scales* | **Constipation** | | | | **Fatigue** | | | | **Pain** | | | |
|  | Score | B | 95% CI | *p* | Score | B | 95% CI | *P* | Score | B | 95% CI | *P* |
| Baseline | 6.4 |  |  |  | 27.1 |  |  |  | 19.5 |  |  |  |
| After 1 month | 22.3 | 16.1 | [7.7, 24.6] | **<0.001** | 44.5 | 17.4 | [10.9, 24.0] | **<0.001** | 38.4 | 18.9 | [11.4, 26.4] | **<0.001** |
| After 3 months | 8.3 | 1.9 | [-6.7, 10.5] | 0.7 | 25.3 | -1.8 | [-8.5, 4.9] | 0.6 | 20.0 | 0.5 | [-7.2, 8.1] | 0.9 |
| Age (reference 70 yr) |  | -0.1 | [-0.5, 0.4] | 0.8 |  | 0.01 | [-0.4, 0.4] | >0.9 |  | -0.2 | [-0.7, 0.2] | 0.3 |
| Sex |  |  |  |  |  |  |  |  |  |  |  |  |
| Female |  |  | Reference |  |  |  | Reference |  |  |  | Reference |  |
| Male |  | -3.5 | [-11.4, 4.4] | 0.4 |  | -6.5 | [-14.0, 1.1] | 0.09 |  | -2.5 | [-10.3, 5.4] | 0.5 |
| pT-stage |  |  |  |  |  |  |  |  |  |  |  |  |
| <pT2 |  |  | Reference |  |  |  | Reference |  |  |  | Reference |  |
| ≥pT2 |  | 2.8 | [-2.5, 8.2] | 0.3 |  | 2.3 | [-3.6, 8.2] | 0.4 |  | -0.8 | [-6.6, 5.0] | 0.8 |
| CCI |  |  |  |  |  |  |  |  |  |  |  |  |
| ≤4 |  |  | Reference |  |  |  | Reference |  |  |  | Reference |  |
| >4 |  | 1.6 | [-4.6, 7.9] | 0.6 |  | 1.2 | [-5.6, 8.0] | 0.7 |  | 1.7 | [-5.0, 8.4] | 0.6 |
| Type of surgery |  |  |  |  |  |  |  |  |  |  |  |  |
| Open |  |  | Reference |  |  |  | Reference |  |  |  | Reference |  |
| Laparoscopic/Robot |  | 1.4 | [-6.1, 8.8] | 0.7 |  | -4.8 | [12.9, 3.4] | 0.3 |  | -3.9 | [-11.9, 4.1] | 0.3 |
| Lymph node dissection |  |  |  |  |  |  |  |  |  |  |  |  |
| No |  |  | Reference |  |  |  | Reference |  |  |  | Reference |  |
| Yes |  | -1.0 | [-8.4, 6.4] | 0.8 |  | -0.3 | [-6.8, 6.3] | 0.9 |  | -2.9 | [-10.0, 4.3] | 0.4 |
| Surgical complication |  |  |  |  |  |  |  |  |  |  |  |  |
| No |  |  | Reference |  |  |  | Reference |  |  |  | Reference |  |
| Yes |  | 3.9 | [-2.4, 10.3] | 0.23 |  | 5.7 | [0.02, 11.3] | 0.050 |  | 5.8 | [-0.4, 11.9] | 0.066 |
| CCI = Charlson Comorbidity Index; CI = Confidence Interval; *p* values of <0.05 are considered significant | | | | | | | | | | | | |

**References**

1. Cocks K, King MT, Velikova G, et al. Evidence-based guidelines for interpreting change scores for the European Organisation for the Research and Treatment of Cancer Quality of Life Questionnaire Core 30. *Eur J Cancer*. Jul 2012;48(11):1713-21. doi:S0959-8049(12)00211-0 [pii] 10.1016/j.ejca.2012.02.059
